# Sodium leak through K2P potassium channels and cardiac arrhythmia, an emerging theme

**DOI:** 10.15252/emmm.201607479

**Published:** 2017-03-03

**Authors:** Steve AN Goldstein

**Affiliations:** ^1^ Department of BiochemistryBrandeis UniversityWalthamMAUSA

**Keywords:** Cardiovascular System, Genetics, Gene Therapy & Genetic Disease

## Abstract

In this issue of *EMBO Molecular Medicine*, Decher *et al* (2017) identify a point mutation in the K2P2 (TREK‐1) potassium (K^+^) channel that changes function in just those ways expected to predispose to right ventricular outflow tract (RVOT) ventricular tachycardia (VT) in the patient they study. Whereas wild‐type channels are selective for K^+^ and inhibited by β‐adrenergic stimulation, mutant I267T channels pass sodium (Na^+^) into the cells, even during β‐adrenergic stimulation, and are more active in response to membrane stretch, changes predicted to enhance cardiac myocyte excitability. The report contributes to accumulating evidence for Na^+^ leak via K2P channels in association with normal development (Thomas *et al*, 2008), acquired arrhythmia (Ma *et al*, 2011), and now a missense mutation. Decher *et al* (2017) both inform and direct us toward interesting opportunities for further investigation.

Each normal heartbeat results from an action potential (AP) wave that begins in the cardiac atria and travels to the ventricles, causing the chambers to contract and pump blood (Fig 1A). Whereas most changes in the rate or rhythm of heartbeats (arrhythmia) can be well tolerated, VT can lead to hemodynamic compromise if the heart cannot supply enough blood to the body (Fig 1B and C), a particular risk if the abnormal rhythm is sustained or degenerates to ventricular fibrillation. Like 10% of patients with VT of unknown cause (idiopathic), the patient in this study has no structural heart disease, no metabolic or electrolyte abnormalities, and no other mutations known to cause arrhythmia. About 70% of idiopathic VT begins in the RVOT. While the prognosis for RVOT‐VT is generally excellent, sudden death can occur and periodic follow‐up is important to rule out latent, progressive arrhythmogenic right ventricular cardiomyopathy (Noda *et al*, 2005). If symptoms or family history argue for treatment, patients respond well to pharmacotherapy or ablation of the RVOT site producing the aberrant electrical signals via a catheter threaded into the heart through a vein. The disorder usually presents between 30 and 50 years (range 6–80), hormonal influences are seen in premenopausal women, and episodes are often triggered by sympathetic stimulation (for example, anxiety, excitement, or caffeine).

**Figure 1:**
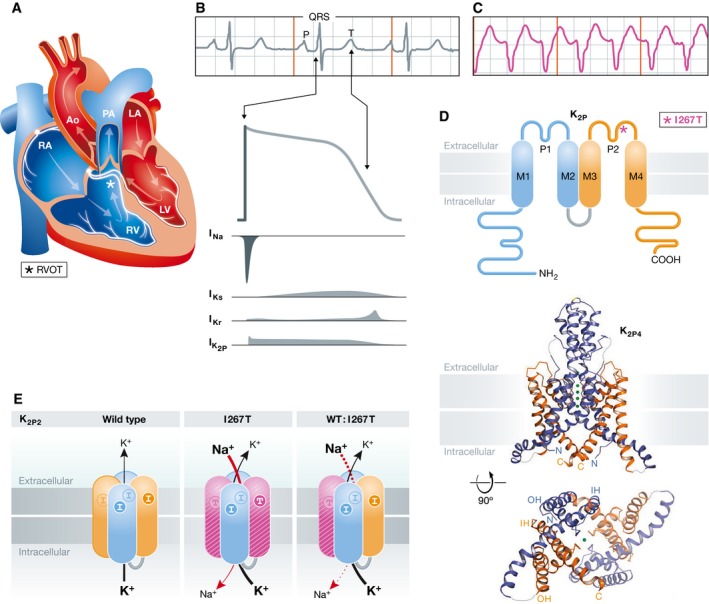
Molecular cardiology of RVOT‐VT: anatomy, electrophysiology, and the structure and function of K2P2 (A) The chambers of the heart and conduction system. Venous blood flows back from the body to the right atrium (RA), is pumped to the right ventricle (RV), and then out through the RVOT (*) into the pulmonary arteries (PA) to the lungs. Oxygenated blood returns to the left atrium (LA), is pumped to the left ventricle (LV), and then out the aorta (Ao) to the body. The conduction system is indicated in silver: excitation begins in the sinoatrial node atop the RA, travels down to the atrioventricular node, and after a brief delay, travels the Purkinje fiber system to excite the ventricles. (B) Normal sinus rhythm and the ventricular AP. Top: surface ECG (lead II) shows the P wave associated with atrial excitation, the QRS reflecting ventricular activation, and the T wave showing ventricular repolarization. The rapid rise in the membrane potential is due to SCN5A (I_Na_) and repolarization is due to K^+^ flux via I_Ks_, I_Kr_, and K2P channels (currents not to scale). (C) Monomorphic VT on a surface ECG. Rapid, repetitive excitations from an abnormal ventricular locus (lead II); the full 12‐lead ECG shows LBBB and an inferior axis, common for RVOT‐VT. (D) K2P subunit topology and channel structure. Top: One K2P subunit has four transmembrane segments, two P loop domains, and intracellular N and C termini; I267T in the second P loop domain is indicated (*). Middle: Ribbon representation of K2P4 with the first P domain in blue, the second in orange, and K^+^ ions as green spheres with membrane boundaries suggested by gray bars, by permission (Brohawn *et al*, 2012). Bottom: A view from the cytoplasm. (E) Three types of K2P2 channels in the patient. One wild‐type (WT) gene and one for I267T is predicted to yield channels with two WT subunits, two I267T subunits (the second P domain is now pink), or one of each. The WT channels pass K^+^ outward. Decher *et al* (2017) show that channels with two I267T subunits pass inward Na^+^; the drawing shows channels with two altered residues, one on each I267T subunit and mixed channels with one altered residue (shown passing Na^+^ but this has not yet been demonstrated).

Decher *et al* (2017) employed a candidate gene approach to identify the mutation and then judged its potential to cause disease by studying its frequency in the population and its impact on channel function. Sequencing *KCNK2*, the gene for K2P2, in 438 patients with idiopathic arrhythmia yielded one patient among the 40 with a diagnosis of RVOT‐VT who was heterozygous for I267T, carrying one wild‐type (WT) gene and one mutant allele. Consistent with a role in disease causation, the change was not seen in 379 controls; it was present in 3 of 13,003 alleles in the Exome Variant Server database; in 388 other genes the patient showed variations judged to be neutral by a suite of pathogenicity prediction tools; and I267T was in a noteworthy location, the second pore loop (P) domain of the K2P2 subunit.

Each K2P channel subunit has two pore (P) loop domains, hence the K2P nomenclature (Fig 1D) (Plant *et al*, 2017). A complete K2P channel has two subunits that create one membrane‐spanning ion conduction pore lined by four P loop domains on the central axis of symmetry (Brohawn *et al*, 2012). Seeking to recapitulate the three K2P2 channels expected to form in the patient (channels with two WT subunits, two I267T subunits, or one of each type, Fig 1E), the authors injected cRNAs into frog oocytes to express the subunits for electrophysiology studies.

The effects of I267T were dramatic. Cells with I267T were different in two key ways from those with only WT channels: net outward currents were decreased and the resting membrane potential (RMP) of the cells more depolarized (positive). Both findings result from inward Na^+^ leak through mutant channels. The WT channels passed outward currents because they conducted K^+^ in preference to Na^+^ and the electrochemical driving force favors efflux of K^+^ down its concentration gradient (K^+^_in_ ~140 mM versus K^+^_out_ ~5 mM) during the cardiac cycle—this is a restoring current that hyperpolarizes the cells toward RMP. In contrast, cells with I267T passed Na^+^ inward, down its concentration gradient (Na^+^_out_ ~135 mM versus Na^+^_in_ ~10 mM), decreasing the net outward current—an excitatory effect. Moreover, the RMP is set by the relative permeability of ions across the plasma membrane, so excess Na^+^ influx caused an excitatory depolarization in the resting set point. In a nice experimental turn, they showed that the magnitude of K^+^ efflux was the same through WT and I267T channels in the absence of external Na^+^; this indicated that the mutation did not alter the synthesis, assembly, trafficking, or half‐life of the channels as a basis for decreased outward current in the oocytes. Unexpectedly, they found I267T channels both resisted β‐adrenergic suppression, allowing Na^+^ leak to persist, and to be more sensitive to activation by membrane stretch than WT.

Each of these effects of I267T is expected to be pro‐arrhythmic *in vivo*. Indeed, they found that overexpression of I267T in human atrial tumor cells depolarized the RMP, increased the rate of spontaneous beating, and slowed the AP upstroke velocity (a parameter known to contribute to ventricular re‐entry arrhythmias), compared to WT. As an encouraging first step toward mechanistically inspired therapy, BL‐1249, a fenamate compound, restored K^+^ selectivity to cells expressing the mutant channels.

The human heart beats ~100,000 times every day, even in patients predisposed to arrhythmia. In such a resilient system, is it reasonable to ascribe RVOT‐VT to leaky K2P channels? Studies of SCN5a and long QT syndrome (LQTS type 3) suggest this is a sensible hypothesis (Belardinelli *et al*, 2015; Bohnen *et al*, 2017). Each AP results from a delicate orchestration of ion fluxes (Fig 1B). In the ventricles, the AP begins when the membrane reaches a threshold potential and SCN5a voltage‐gated Na^+^ channels open to produce an explosive influx of Na^+^ current (I_Na_) leading cells to depolarize. SCN5a channels inactive rapidly, influx of calcium maintains the AP plateau, and the AP ends due to K^+^ efflux through depolarization‐activated I_Kr_ and I_Ks_ channels. Mutations that produce a small excess of I_Na_ through SCN5a channels can seriously disrupt cardiac electrophysiology, prolonging the AP duration, slowing repolarization and, in some cases, increasing cytosolic Na^+^ sufficiently to drive pumps to increase intracellular calcium, all effects that are pro‐arrhythmic. Thus, changes that render SCN5a inactivation slow or incomplete and increase I_Na_ late in the AP from ~0.3–1% of peak current are sufficient to predispose to VT, Torsade de pointe, and sudden death. The fine balance of currents is apparent: the small late Na^+^ current passed by SCN5a under normal conditions is similar in size but opposite in direction to I_Ks_ in guinea pig ventricle.

Other channels, including K2P channels, also contribute to the AP, modifying its shape differentially in the atria, conduction pathways, ventricles, and across the myocardial wall. While the magnitude of K2P2 K^+^ current in human heart cells is unknown, the absence of a prolonged QT in the patient suggests the magnitude of Na^+^ flux via I267T channels in ventricular cells is less than I_Na_ late. This suggests I267T imbalances activating and restoring currents locally in the RVOT perhaps due to increased sensitivity of I267T to membrane stretch, a pro‐arrhythmic stimulus even under wild‐type conditions. Also consistent with a mechanism involving decreased repolarization reserve, as seen with excess inward Na^+^ current, K^+^ channel expression is regulated by sex and stress hormones.

Humans have 15 genes for K2P channels (Plant *et al*, 2017). Identified based on their unique subunit architecture (Ketchum *et al*, 1995; Lesage *et al*, 1996) as well as their functional attributes (Goldstein *et al*, 1996), understanding their roles in physiology is a work in process. Operating across the body as homodimers and heterodimers, K2P channels are responsible for many of the regulated background currents essential to nerve and muscle function because they are open across the physiological voltage range, passing outward K^+^ currents that help set RMP and repolarize the membrane below firing threshold after excitation. It seems logical then that changing the selectivity of cardiac K2P2 channels could impact tissue excitability. While K2P2 is highly expressed in the human brain and endocrine system, transcripts are present in the heart accompanied by those for K2P1, K2P3, K2P5, K2P15, and K2P17. Two prior studies seem particularly germane.

We observed a natural structural change in K2P2 that allows it to conduct Na^+^ (Thomas *et al*, 2008). Thus, K2P2 protein and a variant lacking 56 N‐terminal residues due to alternative translation initiation (K2P2∆56) are expressed at different levels in different regions of the rat CNS in a manner that changes during development. Whereas K2P2 is selective for K^+^, the short version conducts Na^+^ and, similar to I267T, this leads to a depolarized RMP. Thus, Na^+^ flux through K2P2∆56 appears to have normal roles in the rat brain during maturation and adult function. Of note, Decher *et al* (2017) suggest an explanation for a discrepancy in the literature; a group that did not observe Na^+^ flux via K2P2∆56 channels had fenamates (including BL‐1249), that restore K^+^ selectivity to I267T channels, in their solutions.

Ma *et al* (2011) observed Na^+^ flux via the K2P1 (TWIK‐1) channel. Whereas I267T and K2P2∆56 channels show stable changes in ion selectivity, WT K2P1 channels alter their permeation attributes reversibly, allowing Na^+^ to pass when exposed to the low extracellular levels of K^+^ present in the serum of patients with hypokalemia. Na^+^ influx via K2P1 appears to explain arrhythmia‐associated “paradoxical depolarization” that is detected in a subset of cardiac myocytes: whereas lowering serum K^+^ would be expected to increase driving force for K^+^ efflux and lead to membrane hyperpolarization, K2P1 channels lose their preference for K^+^ allowing Na^+^ influx and depolarization. The selectivity of classical K^+^ channels formed by subunits with one P domain appears to be stable under physiological conditions. In contrast, K2P1 and K2P2 are now seen to loosen their restriction against Na^+^ permeation under both natural and disease‐associated conditions. Perhaps, other K2P channels will follow suit.

The findings by Decher *et al* (2017) have begun the process of correlating genotype and phenotype. As each journey begins with a step, they identify one patient with I267T. More patients must be studied to verify the association with RVOT‐VT, and this sentinel case will stimulate that valuable inquiry. Other open questions will thereby be addressed: does I267T act alone or is it linked to changes in other genes or the vast non‐coding regions of the genome? What is the role of ethnic context? Are other variants of KCNK2 pro‐arrhythmic? How do epigenetic influences impact disease penetrance? Do mutations in other genes alter ion selectivity (stably or in response to environmental perturbations) to produce arrhythmia?

We molecular cardiologists employ model systems to assess function but none recapitulates the human heart or its cells with fidelity. Decher *et al* (2017) employ heterologous overexpression and modeling to achieve detailed biophysical characterization of I267T but elevated channel levels and non‐natural cellular milieus are limitations. Open questions therefore include where, when, and with what partners does K2P2 operate in the human heart (a conundrum for channels that have been studied for decades longer)? Does K2P2 act similarly in myocytes at low natural levels? Does I267T in the brain influence cardiac function? An approach to some of these issues is suggested by rapid advances in CRISPR‐Cas9 technology: expressing I267T from the native K2P2 locus might reveal susceptibility to RVOT‐VT in an animal, show native Na^+^ influx, membrane depolarization, and calcium overload in cardiomyocytes, and permit appraisal of fenamates as drug leads.

Decher and colleagues increase our understanding of normal cardiac physiology, the operation of K2P channels, and the pathophysiology of RVOT‐VT. The work is a step toward transforming an idiopathic syndrome into defined disorders with explicit causality. Actualization of this aspect of precision medicine will improve evidence‐based interventions and serve as the basis for risk stratification. This is particularly important for unpredictable, multifactorial disorders like episodic arrhythmias where genetic variation predisposes to events that are infrequent. Thus, genomic sequencing may someday flag a person with a K2P Na^+^ leak variant, and indicate if she is likely to respond to a BL1249 derivative, before experiencing a frightening arrhythmia. More urgently, this type of linkage may inform physicians when it is critical to intervene. Rare patients who die suddenly with a diagnosis of RVOT‐VT do not present differently than the large majority who have a benign outcome and, yet, an aggressive approach to all patients is ill advised because RVOT‐VT is common and therapies carry their own risks (Noda *et al*, 2005). Thus, studies of cells, animals, and patients to validate the linkage of I267T and RVOT‐VT, identify other changes that predispose to RVOT‐VT, and place the role of abnormal Na^+^ flux into a diagnostic and therapeutic context will build on this elegant work to advance the frontiers of molecular medicine.
